# Integrated transcriptomic and metabolomic profiling reveals dysregulation of purine metabolism during the acute phase of spinal cord injury in rats

**DOI:** 10.3389/fnins.2022.1066528

**Published:** 2022-11-23

**Authors:** Zhong Zeng, Mei Li, Zhanfeng Jiang, Yuanxiang Lan, Lei Chen, Yanjun Chen, Hailiang Li, Jianwen Hui, Lijian Zhang, Xvlei Hu, Hechun Xia

**Affiliations:** ^1^Department of Neurosurgery, General Hospital of Ningxia Medical University, Yinchuan, China; ^2^Ningxia Key Laboratory of Stem Cell and Regenerative Medicine, General Hospital of Ningxia Medical University, Yinchuan, China; ^3^School of Clinical Medicine, Ningxia Medical University, Yinchuan, China; ^4^Ningxia Key Laboratory of Craniocerebral Diseases, Ningxia Medical University, Yinchuan, China; ^5^Department of Neurosurgery, The First People’s Hospital of Shizuishan, Shizuishan, China; ^6^Department of Neurosurgery, Affiliated Hospital of Hebei University, Hebei University, Baoding, China; ^7^Department of Neurosurgery, Shanxi Provincial People’s Hospital, Taiyuan, China

**Keywords:** spinal cord injury, transcriptomics, metabolomics, purine metabolism, integrated analysis

## Abstract

**Introduction:**

Spinal cord injury (SCI) results in drastic dysregulation of microenvironmental metabolism during the acute phase, which greatly affects neural recovery. A better insight into the potential molecular pathways of metabolic dysregulation by multi-omics analysis could help to reveal targets that promote nerve repair and regeneration in the future.

**Materials and methods:**

We established the SCI model and rats were randomly divided into two groups: the acute-phase SCI (ASCI) group (*n* = 14, 3 days post-SCI) and the sham group with day-matched periods (*n* = 14, without SCI). In each group, rats were sacrificed at 3 days post-surgery for histology study (*n* = 3), metabolome sequencing (*n* = 5), transcriptome sequencing (*n* = 3), and quantitative real-time polymerase chain reaction (*n* = 3). The motor function of rats was evaluated by double-blind Basso, Beattie, and Bresnahan (BBB) Locomotor Scores at 0, 1, 2, 3 days post-SCI in an open field area. Then the transcriptomic and metabolomic data were integrated in SCI model of rat to reveal the underlying molecular pathways of microenvironmental metabolic dysregulation.

**Results:**

The histology of the microenvironment was significantly altered in ASCI and the locomotor function was significantly reduced in rats. Metabolomics analysis showed that 360 metabolites were highly altered during the acute phase of SCI, of which 310 were up-regulated and 50 were down-regulated, and bioinformatics analysis revealed that these differential metabolites were mainly enriched in arginine and proline metabolism, D-glutamine and D-glutamate metabolism, purine metabolism, biosynthesis of unsaturated fatty acids. Transcriptomics results showed that 5,963 genes were clearly altered, of which 2,848 genes were up-regulated and 3,115 genes were down-regulated, and these differentially expressed genes were mainly involved in response to stimulus, metabolic process, immune system process. Surprisingly, the Integrative analysis revealed significant dysregulation of purine metabolism at both transcriptome and metabolome levels in the acute phase of SCI, with 48 differential genes and 16 differential metabolites involved. Further analysis indicated that dysregulation of purine metabolism could seriously affect the energy metabolism of the injured microenvironment and increase oxidative stress as well as other responses detrimental to nerve repair and regeneration.

**Discussion:**

On the whole, we have for the first time combined transcriptomics and metabolomics to systematically analyze the potential molecular pathways of metabolic dysregulation in the acute phase of SCI, which will contribute to broaden our understanding of the sophisticated molecular mechanisms of SCI, in parallel with serving as a foundation for future studies of neural repair and regeneration after SCI.

## Introduction

Spinal cord injury (SCI) will substantially damage the central nervous system due to its devastating property, which is usually a direct injury caused by trauma and other factors (O’Shea et al., 2017). Patients with SCI suffer motor and sensory function loss at various degrees due to disruption of the neural circuit, and in severe cases, even complete loss of neurological function, which seriously affects the living quality of both patients and their families and increases the economic burden on society (Hachem et al., 2017; Alizadeh et al., 2019). Although numerous researchers have made great efforts to explore neuroprotective and neuroregenerative treatments to completely improve the neurological function of patients, there is still no treatment available to cure patients with SCI due to the complex pathophysiological mechanisms (Ahuja and Fehlings, 2016; Anjum et al., 2020).

According to the pathophysiological process, SCI can be classified into primary injury and secondary injury (Ahuja et al., 2017). The former mechanical injury triggers a variety of interacting molecular pathological cascades that disrupt the microenvironment of the lesion area, which in turn facilitates the development of secondary SCI (Anjum et al., 2020; Baroncini et al., 2021). During the secondary SCI phase, the balance of the spinal cord microenvironment at the molecular, cellular and tissue levels is disrupted: the molecular level mainly involves the production of lipid mediators and free radicals, the expression of neurotrophic factors and various cytokines and chemokines; the cellular level involves activation of microglia and astrocytes, differentiation of endogenous neural stem cells and oligodendrocyte progenitors, and infiltration of macrophages and immune cells; hemorrhage and ischemia, demyelination and remyelination, vascular remodeling, and glial scar formation will mainly occur at the tissue level (Fan et al., 2018; Bradbury and Burnside, 2019; Lindsay et al., 2020). The disruption of these balances is accompanied by severe metabolic dysregulation that greatly hinders nerve regeneration and functional recovery after SCI (Sullivan et al., 2007; Fujieda et al., 2012), thus understanding the underlying molecular mechanisms affecting the disruption of the microenvironment in the injured spinal cord contributes to the development of more effective neuroprotective measures.

With the advancement of high-throughput technologies, a wide spectrum of “omics” techniques have been derived, including genomics, transcriptomics, proteomics, and metabolomics. Researchers have gained insight into the molecular mechanisms of disease development through the use of genomics, transcriptomics, and proteomics (Karahalil, 2016; Olivier et al., 2019; Chu et al., 2021). Furthermore, as researchers deepen their research on metabolites, the application of metabolomics has gradually started to increase. Being the end products of cellular activities, metabolites are closer to the phenotype, and subtle changes in genes and proteins are amplified in metabolites, which is a unique advantage of metabolomics. Currently, metabolomics is increasingly used as an analytical tool for early diagnosis, prognostic assessment, and monitoring of treatment response in cancer and metabolic diseases (Bansal et al., 2022; di Meo et al., 2022). On the same note, there is a growing interest in the role of metabolomics in SCI, especially in studies related to specific biomarkers for the assessment of SCI severity and prognosis (Jiang et al., 2010; Peng et al., 2014; Wu et al., 2016; Yang et al., 2022). Metabolomics also facilitates insight into the pathophysiological processes of disease and the alteration of metabolic pathways *in vivo* to study the overall stress of the organism in response to disease or environmental stimuli (Mathew et al., 2019; Gao et al., 2022). In SCI, metabolomics approaches to identify changes in relevant metabolites after the injury can help to understand and further explore the pathophysiological mechanisms of secondary SCI (Figueroa and De Leon, 2014; Peng et al., 2014).

However, it is difficult for any single “omics” to fully reveal the process of disease development. Metabolomics studies the terminus of the metabolic process of substances in the body and needs to be combined with other “omics,” such as transcriptomics, to further elucidate the pathophysiological process of diseases systematically. As a result, an increasing number of studies have begun to shift from a single “omics” to the use of “multi-omics” to describe the development of disease, which can effectively bridge the insufficiency of a single “omics” and is likely to unravel much more of the biology than the total of the separate analysis. In terms of SCI, most of the studies are currently based on a single “omics” (Yang et al., 2021; Yao et al., 2021; Rodgers et al., 2022), and the application of integrated “multi-omics” to study the pathophysiological processes of SCI needs to be further improved.

In the present study, to further systematically explore the dysregulation of microenvironmental metabolism and identify the potential molecular pathways in SCI, we performed transcriptome and metabolome sequencing as well as bioinformatics analysis of the injured spinal cord during the acute phase and the integrative analysis of transcriptomic and metabolomic data for the first time in SCI.

## Materials and methods

### Animals and groups

A total of 28 adult male specific pathogen-free Sprague-Dawley (SD) rats, weighing 220–250 g, were purchased from the Experimental Animal Center of Ningxia Medical University (approval number: 2017-073). Rats were housed in individually ventilated cages and placed in a standard environment (12 h light/dark cycle) with food and water freely available and then were acclimated to the environment for 1 week before surgery. Rats were randomly divided into two groups: the acute-phase SCI (ASCI) group (*n* = 14, 3 days post-SCI) and the sham group with day-matched periods (*n* = 14, without SCI). In each group, rats were sacrificed at 3 days post-surgery for histology study (*n* = 3), metabolome sequencing (*n* = 5), transcriptome sequencing (*n* = 3), and quantitative real-time polymerase chain reaction (qPCR) (*n* = 3). All experimental procedures were approved by the Ningxia Medical University Animal Ethics Committee.

### Rat spinal cord injury model

To establish the SCI model, rats were anesthetized by intraperitoneal injection of 1% pentobarbital (5 mg/100 g), and body temperature was maintained with a heating pad. After locating the 9th thoracic spinal vertebra (T9)-T10, the surgical area was thoroughly disinfected with iodophor and 75% ethanol following skin preparation, and the skin, subcutaneous tissues, fascia, and muscles were incised in the dorsal midline layer by layer. Then, the paravertebral muscles were stripped along the spinous process to both sides, the T8-T11 spinous processes and the vertebral plates were exposed and then removed to fully expose T10 for lateral hemisection which was established by inserting a 1 ml syringe needle vertically along the middle spinal artery and then slicing outward through the left half of the spinal cord three times to make sure that the hemisection is completed. The sham group only received laminectomy without injuring the spinal cord. After the surgery, the animals were well taken care of and given a bladder massage three times a day to assist in urination. The motor function of rats was evaluated by double-blind Basso, Beattie, and Bresnahan (BBB) Locomotor Scores (Basso et al., 1995) at 0, 1, 2, 3 days post-SCI in an open field area.

### Hematoxylin-eosin and Nissl staining

Rats from sham (*n* = 3) and ASCI (*n* = 3) groups were anesthetized by intraperitoneal injection of 1% pentobarbital (5 mg/100 g), and transcardially perfused with 0.9% saline (∼300 ml, ambient temperature), followed by buffered 4% formaldehyde solution. The spinal cord segment (about 2 cm) was taken with the injury site as the midpoint, fixed in 4% formaldehyde for 24 h, then embedded with alcohol gradient dehydration and paraffin. The horizontal sections of the spinal cord were cut and stained with hematoxylin-eosin (HE) and Nissl staining for histopathology examination under a light microscope (Nikon Ci-L, Japan).

### Metabolites extraction and sequencing

A total of 10 mg of the injured spinal cord sample was weighted to an Eppendorf tube, and a 1000 μl extract solution (methanol: water = 3: 1, with isotopically labeled internal standard mixture) was added. Samples were homogenized at 35 Hz for 4 min and sonicated for 5 min in an ice water bath after vortex 30 s. The homogenization and sonication cycle was repeated three times. Afterward, the samples were incubated for 1 h at −40°C and centrifuged at 12,000 rpm for 15 min at 4°C. The resulting supernatant was transferred to a fresh glass vial for analysis. The quality control (QC) sample was prepared by mixing an equal aliquot of the supernatants of all the samples. Liquid chromatography-tandem mass spectrometry (LC-MS/MS) were performed using a ultra high performance liquid chromatography (UHPLC) system (Vanquish, Thermo Fisher Scientific, Waltham, MA, USA) with a UPLC HSS T3 column (2.1 mm × 100 mm, 1.8 μm) coupled to Q Exactive HFX mass spectrometer (Orbitrap MS, Thermo Fisher Scientific, Waltham, MA, USA). The mobile phases comprised 5 mmol/L ammonium acetate and 5 mmol/L acetic acid in water (A) and acetonitrile (B). The autosampler temperature was 4°C and the injection volume was 3 μl. The QE HFX mass spectrometer was capable of acquiring MS/MS spectra in information-dependent acquisition (IDA) mode under the control of acquisition software (Xcalibur, Thermo Fisher Scientific, Waltham, MA, USA). The electrospray ionization source conditions were set as follows: sheath gas flow rate: 30 Arb, auxiliary gas flow rate: 10 Arb, capillary temperature: 350°C, full MS resolution: 60,000, MS/MS resolution: 7,500, collision energy: 10/30/60 in NCE mode, and spray voltage: 4.0 kV (positive) or –3.8 kV (negative).

The raw data were converted to the mzXML format using ProteoWizard and processed with an in-house program, which was developed using R and based on XCMS, for peak detection, extraction, alignment, and integration. Then an in-house MS2 database was applied in metabolite annotation. The cutoff for annotation was set at 0.3.

### Ribonucleic acid extraction and sequencing

Total RNA was extracted from T10 spinal cord segments of rats in ASCI and sham groups by TRIzol reagent (Life Technologies). RNA concentration was measured using NanoDrop 2,000 (Thermo Fisher Scientific, Waltham, MA, USA). RNA integrity was assessed using the RNA Nano 6,000 Assay Kit of the Agilent Bioanalyzer 2,100 system (Agilent Technologies, CA, USA). A total amount of 1 μg RNA per sample was used as input material for the RNA sample preparations. Sequencing libraries were generated using NEBNext^®^ Ultra™ RNA Library Prep Kit for Illumina^®^ (NEB, USA) following the manufacturer’s recommendations and index codes were added to attribute sequences to each sample. Briefly, mRNA was purified from total RNA using poly-T oligo-attached magnetic beads. Fragmentation was carried out using divalent cations under elevated temperature in NEBNext First Strand Synthesis Reaction Buffer (5X). First strand cDNA was synthesized using random hexamer primer and M-MuLV Reverse Transcriptase. Second strand cDNA synthesis was subsequently performed using DNA Polymerase I and RNase H. Remaining overhangs were converted into blunt ends *via* exonuclease/polymerase activities. After adenylation of three’ ends of DNA fragments, NEBNext Adaptor with hairpin loop structure was ligated to prepare for hybridization. To select cDNA fragments of preferentially 240 bp in length, the library fragments were purified with the AMPure XP system (Beckman Coulter, Beverly, MA, USA). Then 3 μl USER Enzyme (NEB, USA) was used with size-selected, adaptor-ligated cDNA at 37°C for 15 min followed by 5 min at 95°C before PCR. Then PCR was performed with Phusion High-Fidelity DNA polymerase, Universal PCR primers, and Index (X) Primer. At last, PCR products were purified (AMPure XP system) and library quality was assessedll2, on the Agilent Bioanalyzer 2,100 system. The clustering of the index-coded samples was performed on a cBot Cluster Generation System using TruSeq PE Cluster Kit v3-cBot-HS (Illumia) according to the manufacturer’s instructions. After cluster generation, the library preparations were sequenced on an Illumina Hiseq platform and paired-end reads were generated.

Raw data of fastq format were firstly processed through in-house perl scripts. In this step, clean data (clean reads) were obtained by removing reads containing adapter, reads containing ploy-N and low-quality reads from raw data. At the same time, Q20, Q30, GC-content, and sequence duplication levels of the clean data were calculated. All the downstream analyses were based on clean data with high quality. The adaptor sequences and low-quality sequence reads were removed from the data sets. Raw sequences were transformed into clean reads after data processing. These clean reads were then mapped to the reference genome sequence. Only reads with a perfect match or one mismatch were further analyzed and annotated based on the reference genome. Tophat2 tools soft were used to map with the reference genome.

### Bioinformatics analysis

To further investigate the biological significance of the results of RNA and metabolites sequencing, we performed bioinformatics analysis. The databases used for gene functional annotation were the National Center for Biotechnology Information non-redundant protein sequence database (NR); the database of Homologous protein family (Pfam); the database of Clusters of Orthologous Groups of proteins (COG); the database of Clusters of Protein homology (KOG); a manually annotated, non-redundant protein sequence database (Swiss-Prot); GO (Gene Ontology database) and the database of Kyoto Encyclopedia of Genes and Genomes (KEGG). Briefly, Coding Potential Calculator two was used to search for unigene with coding potential, and the unearthed codable unigene was sequenced against NR, Swiss-Prot, GO, COG, KOG, KEGG, and Pfam databases using BLAST software to obtain annotation information. GO enrichment analysis of the differentially expressed genes (DEGs) was implemented by the GOseq R packages based on Wallenius non-central hyper-geometric distribution. For pathway enrichment analysis of DEGs and differentially expressed metabolites (DEMs), we used KEGG and MetaboAnalyst^1^ databases.

### Integrative analysis of the transcriptomic and metabolomic data

To obtain further insights into the metabolic disturbance of ASCI from different biological functional levels, we performed an integrative analysis to determine the pathways related to genes and metabolites by MetaboAnalyst Joint-Pathway Analysis. Only pathways with false discovery rate (FDR) < 0.01, and numbers of both genes and metabolites > 3 were considered as significantly altered. The KEGG pathway of integrative analysis was plotted using the OmicStudio tools at https://www.omicstudio.cn/tool.

### Quantitative real-time polymerase chain reaction assay

We performed a qPCR assay to validate the gene expression changes obtained from the transcriptomic analysis. The primer sequences were shown in Table 1. Total RNA of the injured spinal cord was extracted with TRIzol reagent. Thermo Nanodrop Analyzer and Light Cycle 96 Real-time PCR system were used to assess the quality of RNA and to perform reverse transcription, respectively. Relative expression of genes was determined using the 2^–ΔΔ*Ct*^ method using glyceraldehyde-3-phosphate dehydrogenase (GAPDH) as an internal reference. The gene expression was tested in three rats and each sample was tested in triplicate.

**TABLE 1:** Forward and reverse primer sequences for quantitative real-time polymerase chain reaction (qPCR).

Gene	Forward	Reverse
Tmsb4x	CCTTCTATCCTTCCCTGCCTCTCC	TTCCCTTCCCGTTACTCAGATACCC
Spp1	GACGATGATGACGACGACGATGAC	GTGTGCTGGCAGTGAAGGACTC
Rplp0	GCGGTAGGACGGATGAGAGGAG	GGTGTTCTGAGCAGGTACTGTGAC
Ftl1	AACCACCTGACCAACCTCCGTAG	CAAAGAGATACTCGCCCAGAGATGC
Plp1	GGTCTGCCTCCACTCACTCCTC	TCTGCCCAGTCATTAGCCCTCTAC
GAPDH	AGACAGCCGCATCTTCTTGT	CTTGCCGTGGGTAGAGTCAT

### Data analysis

In the present study, BBB score data were analyzed in GraphPad Prism 8.01 (GraphPad Software, San Diego, CA, USA) and are presented as the mean ± standard deviation (SD). The student’s *t*-test was used for statistical analysis and *P* < 0.05 was considered statistically significant. For transcriptomics, the differential expression analysis of ASCI and sham groups was performed using the DESeq R package (1.10.1). The resulting *p*_adj_ (adjusted) values were adjusted using Benjamini and Hochberg’s approach for controlling the FDR. Genes with an *P*_adj_ < 0.05 found by DESeq were assigned as differentially expressed. For metabolomics, we used the SIMCA16.0.2 software package (Sartorius Stedim Data Analytics AB, Umea, Sweden) for multivariate analysis. Orthogonal projections to latent structures-discriminant analysis (OPLS-DA) was carried out to visualize group separation and find significantly changed metabolites based on variable importance in the projection (VIP) >1 and *P* < 0.05.

## Results

### Histological alterations of the microenvironment and reduction of locomotor function in acute-phase spinal cord injury

**FIGURE 1:**
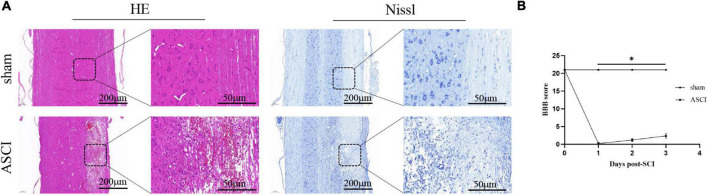
Motor function and histology results during the acute-phase spinal cord injury. **(A)** HE and Nissl staining of the spinal cord horizontally after spinal cord injury. **(B)** Motor function deficits after spinal cord injury were assessed by Basso, Beattie, and Bresnahan Locomotor Scores (data were presented as means ± standard deviation, *n* = 14, Student’s unpaired *t*-test at different time point, *P < 0.05, sham vs. the acute-phase spinal cord injury). Scale bar = 200/50 μm, applicable to all corresponding sections. BBB, Basso, Beattie, and Bresnahan Locomotor Scores; ASCI, the acute-phase spinal cord injury.

In the ASCI group, HE staining showed that the gray matter and white matter in the injury area were poorly demarcated and structurally disorganized, with a large number of cavities and necrotic tissues, enlarged neuronal gaps, neuron reduction or disappearance, glial cells migrating to the injury area and being phagocytosed, and obvious hemorrhagic foci were visible; Nissl staining showed that the Nissl bodies were lightened or disappeared, and the nuclei were fixed and smaller. In the sham group, HE staining showed clear demarcation of white and gray matter in the corresponding area of the spinal cord, with normal structure, the outline of neurons was clear, and neurons and glial cells were evenly distributed; Nissl staining showed that the nuclei of neuronal cells were large, the nucleoli were obvious and abundant, and the Nissl bodies in the cytoplasm were clear (Figure 1A). The BBB score for the ASCI group was badly decreased in comparison to the sham group, which implies a severe reduction in locomotor function following SCI (all *P* < 0.05; Figure 1B).

### Metabolic dysregulation of the microenvironment in acute-phase spinal cord injury

**FIGURE 2:**
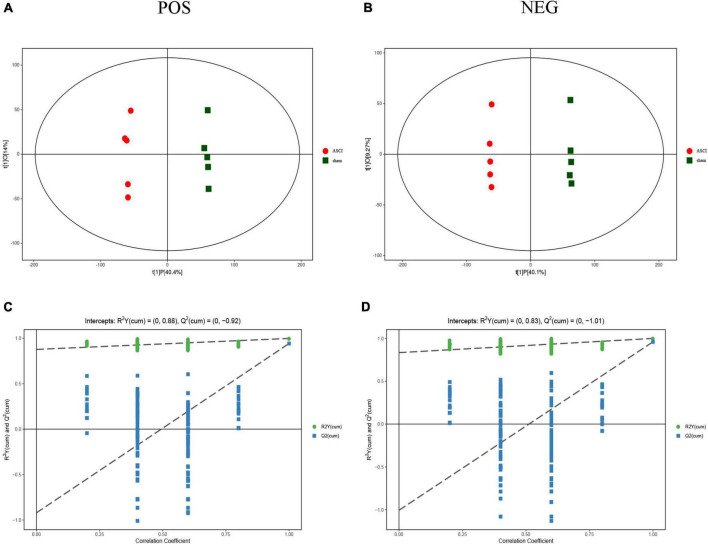
Score scatter plot of orthogonal projections to latent structures-discriminant analysis (OPLS-DA) model and permutation test of OPLS-DA model for group sham vs. the acute-phase spinal cord injury in both positive ion mode and negative ion mode. **(A,B)** OPLS-DA model, horizontal axis indicates the predicted principal component scores of the first principal component, demonstrating between-sample group differences, and vertical axis indicates the orthogonal principal component scores, demonstrating within-sample group differences, with each scatter representing a sample and the scatter shape and color indicating different experimental groupings. **(C,D)** Permutation test of OPLS-DA model shows that the model is good and no overfitting phenomenon. POS, positive ion mode; NEG, negative ion mode; ASCI, the acute-phase spinal cord injury.

**FIGURE 3:**
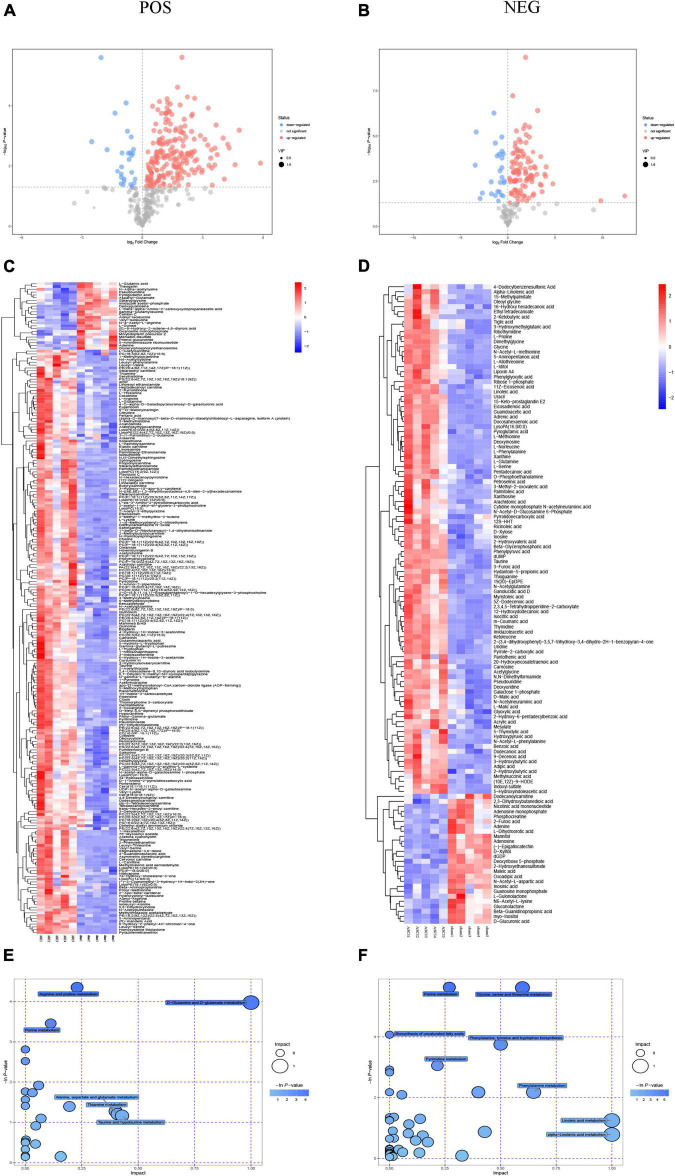
Metabolomic results for group sham vs. the acute-phase spinal cord injury in both positive ion mode and negative ion mode. **(A,B)** Volcano plot in both positive ion mode and negative ion mode. Each dot represents a metabolite, with significantly upregulated metabolites are indicated in red, significantly down-regulated metabolites are indicated in blue, and non-significantly different metabolites are in gray based on variable importance in the projection > 1 and *P* < 0.05. **(C,D)** Heatmap of hierarchical clustering analysis in both positive ion mode and negative ion mode. The horizontal axis represents different experimental groups, the vertical axis represents the differential metabolites compared in that group, the color blocks at different positions represent the relative expression of metabolites in the corresponding position, red color indicates that the substance is highly expressed in the group, blue color indicates that the substance is lowly expressed in the group. **(E,F)** Pathway analysis in both positive ion mode and negative ion mode. Each bubble in the bubble diagram represents a metabolic pathway, the horizontal axis where the bubble is located and the bubble size indicate the size of the influence factor of that pathway in the analysis, and the vertical axis where the bubble is located and the bubble color indicate the *P*-value of the enrichment analysis (taking the negative natural logarithm, namely −ln(p)). POS: positive ion mode; NEG, negative ion mode; ASCI, the acute-phase spinal cord injury.

The results of the OPLS-DA analysis showed that the two sample groups in positive ion mode (POS) and negative ion mode (NEG) were differentiated and validated by permutation test (Figures 2A–D). Metabolomics analysis showed that there were 232 DEMs annotated in the ASCI group compared to the sham group in POS, of which 208 DEMs were significantly upregulated and 24 DEMs were significantly downregulated and there were 128 DEMs annotated in the ASCI group compared to the sham group in NEG, of which 102 DEMs were significantly upregulated and 26 DEMs were significantly downregulated based on VIP > 1 and *P* < 0.05 (Figures 3A,B). Hierarchical clustering analysis revealed that DEMs in the ASCI group were up- and downregulated compared to the sham group in both POS and NEG (Figures 3C,D). To further analyze the metabolic pathways in which DEMs are involved, we performed KEGG pathway enrichment analysis. In POS, a total of 24 metabolic pathways were enriched, of which arginine and proline metabolism, D-glutamine and D-glutamate metabolism, and purine metabolism were significantly changed (Figure 3E) while in NEG, a total of 41 metabolic pathways were enriched, of which purine metabolism, glycine, serine, and threonine metabolism, biosynthesis of unsaturated fatty acids, phenylalanine, tyrosine and tryptophan biosynthesis, pyrimidine metabolism were significantly changed (Figure 3F) based on *P* < 0.05. The top fifteen upregulated DEMs are listed in Table 2. The top fifteen downregulated DEMs are listed in Table 3. All these findings indicate that there was a significant dysregulation of the metabolism in the microenvironment during ASCI.

**TABLE 2:** The top fifteen upregulated differentially expressed metabolites (DEMs).

Metabolite	Log2 fold change	VIP	*P-Value*
Pantothenic acid	12.371	1.282	0.0211
2-Ketobutyric acid	9.837	1.296	0.0388
Leucyl-Serine	4.812	1.559	0.0006
L-Methionine	4.762	1.328	0.0045
Trigonelline	4.609	1.525	0.0091
Diethylcarbamazine N-oxide	4.310	1.552	0.0025
Valyl-Serine	4.240	1.305	0.0019
Guanidoacetic acid	4.061	1.571	0.0005
Isoleucyl-Alanine	4.014	1.555	0.0012
Deoxyinosine	3.996	1.558	0.0017
2,3,4,5-Tetrahydropiperidine-2-carboxylate	3.911	1.268	0.0013
Xanthosine	3.629	1.50	0.0122
Alanyl-Arginine	3.611	1.549	0.0026
Sphingosine	3.601	1.450	0.0093
Indoxyl sulfate	3.537	1.297	0.0054

**TABLE 3:** The top fifteen downregulated differentially expressed metabolites (DEMs).

Metabolite	Log2 fold change	VIP	*P-Value*
Adenosine	–3.440	1.455	0.0004
Adenine	–2.927	1.362	0.0292
Adenosine monophosphate	–2.693	1.362	0.0145
Deoxyguanosine	–2.511	1.523	0.0016
N-a-Acetyl-L-arginine	–1.747	1.456	0.0029
Nicotinic acid mononucleotide	–1.745	1.498	0.0035
Phosphocreatine	–1.729	1.365	0.0160
2-Hydroxyethanesulfonate	–1.463	1.520	5.41E−07
Oxoadipic acid	–1.314	1.527	2.47E−05
N6-Acetyl-L-lysine	–1.312	1.356	0.0200
Molybdopterin precursor Z	–1.230	1.073	0.0149
Inosinic acid	–1.068	1.110	0.0120
Guanosine monophosphate	–1.060	1.001	0.0388
5-Aminoimidazole ribonucleotide	–1.043	1.166	0.0184
Menadiol disulfate	–1.027	1.279	0.0108

### Gene regulation of the microenvironment in acute-phase spinal cord injury

**FIGURE 4:**
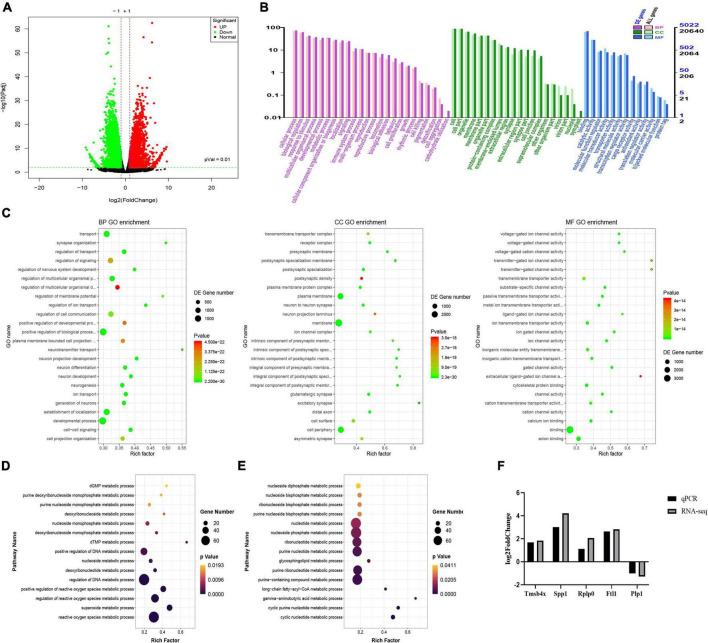
Transcriptome changes during the acute-phase spinal cord injury. **(A)** Changes in genes between the sham and acute-phase spinal cord injury groups. Compared with the sham group, red indicates significant upregulation, green indicates significant downregulation, and black indicates no significant change in the acute-phase spinal cord injury, based on *P* < 0.01. **(B)** Gene ontology annotation classification statistics chart. The horizontal axis is the gene ontology classification, the vertical axis is the percentage of the number of genes on the left and the number of genes on the right. **(C)** The top 20 pathways enriched for differentially expressed genes in biological process, cellular component and molecular function are shown in order. **(D)** Metabolic processes involved in upregulated genes. The vertical axis is the name of the metabolic processes, and the horizontal axis is the number of genes annotated to that metabolic process and its number as a proportion of the total number of genes annotated on it. **(E)** Metabolic processes involved in downregulated genes. **(F)** Quantitative real-time polymerase chain reaction validation of transcriptome data. The result verified that the expression trends of the five genes selected randomly were consistent with the transcriptomic data, where Tmsb4x, Spp1, Rplp0, Ftl1 were upregulated and Plp1 was downregulated. GO, gene ontology; BP, biological process; CC, cellular component; MF, molecular function; QPCR, quantitative real-time polymerase chain reaction; Rplp0, ribosomal protein lateral stalk subunit P0; Tmsb4x, thymosin beta 4, X-linked; Ftl1, ferritin light chain 1; Spp1, secreted phosphoprotein 1; Plp1, proteolipid protein 1.

Transcriptomic analysis showed that there were 5963 DEGs in the ASCI group compared to the sham group, of which 2,848 genes were significantly upregulated and 3,115 genes were significantly downregulated based on fold change ≥2 and *P* < 0.01 (Figure 4A). To further analyze the transcriptome data, we performed GO and KEGG enrichment analysis. The GO analysis includes three main branches, namely biological process (BP), cellular component (CC), and molecular function (MF). The result showed that the DEGs were significantly enriched in 790 BP, 177 CC, and 123 MF based on qvalue < 0.01. The top 20 GO terms of each branch were shown in bubble charts (Figure 4C). In terms of BP, the DEGs were enriched in metabolic process, response to stimulus, immune system process (Figure 4B). Specifically, there were many upregulated DEGs enriched in nucleoside and nucleotide metabolic processes, reactive oxygen species (ROS) metabolic processes, and so forth (Figure 4D); for the downregulated DEGs, there were many enriched in nucleotide metabolic processes and neurotransmitter metabolic processes, and so forth (Figure 4E). Table 4 lists the top twenty upregulated DEGs. Table 5 lists the top twenty downgraded DEGs.

**TABLE 4:** The top twenty upregulated differentially expressed metabolites (DEGs).

Gene	Log2 fold change	*P*_adj_
Hmga2	6.213	3.68E−63
Spp1	4.214	3.05E−57
LOC120101677	6.197	4.82E−55
Hk3	5.702	3.29E−40
LOC120094652	2.906	8.25E−37
Fam111a	4.991	4.89E−36
Ncf2	3.921	5.73E−36
LOC120102105	3.968	1.18E−33
Ngfr	3.203	2.21E−33
LOC120099695	3.974	5.97E−33
Tagln	3.358	2.16E−32
Adam8	4.670	2.72E−32
Btk	2.730	3.62E−31
Mcm6	3.179	4.05E−31
Tcf19	3.413	5.63E−31
Crym	3.564	9.59E−31
Rn18s	4.216	1.42E−30
Mad2l1	2.686	5.71E−30
Fcgr3a	3.596	8.08E−30
Lgmn	2.573	8.95E−30

**TABLE 5:** The top twenty downgraded differentially expressed metabolites (DEGs).

Gene	Log2 fold change	*P*_adj_
Adam11	–3.927	8.68E−62
Ptprn2	–3.883	1.22E−56
Cadps	–3.765	1.03E−54
Cntnap4	–4.030	6.99E−51
Btbd17	–4.394	3.26E−50
Hrh3	–3.360	5.69E−45
Map3k9	–2.562	7.16E−40
Aifm3	–2.443	9.29E−38
Hebp2	–2.715	1.76E−35
Pacsin1	–3.487	1.26E−34
Epn3	–3.006	1.43E−34
Ccdc184	–3.995	1.44E−33
Lgi2	–3.697	1.44E−33
Palm3	–3.323	2.12E−33
Ncs1	–3.483	3.50E−33
Kcnh2	–2.780	6.67E−33
Mctp1	–4.285	4.86E−32
Rapgefl1	–3.913	9.49E−31
Htr7	–3.222	1.41E−30
Kcnab2	–2.838	1.70E−30

Five genes were randomly selected to verify the accuracy of the transcriptome data by qPCR. While the fold change in genes of the two data sets was different, the results of qPCR showed that the genes trended consistently with the transcriptome data. The result showed that the expression of Rplp0 (ribosomal protein lateral stalk subunit P0), Tmsb4x (thymosin beta 4, X-linked), Ftl1 (ferritin light chain 1) and Spp1 (secreted phosphoprotein 1) was upregulated and Plp1 (proteolipid protein 1) was downregulated, confirming the validity of the transcriptome data (Figure 4F).

### Integrative analysis reveals the dysregulation of the purine metabolism in the microenvironment during acute-phase spinal cord injury

**FIGURE 5:**
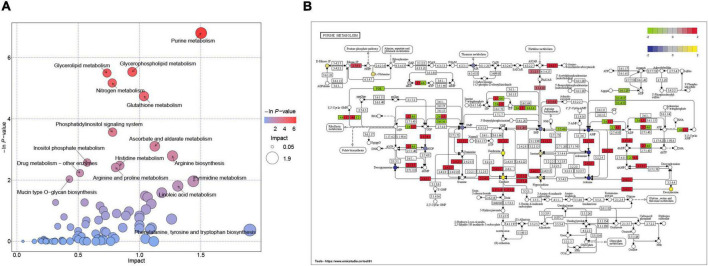
Integration analysis of transcriptomics and metabolomics. **(A)** Pathways enriched with both genes and metabolites. Each bubble in the bubble diagram represents a pathway, the horizontal axis where the bubble is located and the bubble size indicate the size of the influence factor of that pathway in the analysis, and the vertical axis where the bubble is located and the bubble color indicate the *P*-value of the enrichment analysis (taking the negative natural logarithm, namely −ln(p)). **(B)** The molecular mechanisms of dysregulation of purine metabolism at the gene and metabolite levels. Circles represent metabolites and colored ones represent differential metabolites, and rectangles represent genes and colored ones represent differential genes.

To further explore the relationship between transcriptomic and metabolomic changes during ASCI, an integrative analysis was performed using MetaboAnalyst Joint-Pathway Analysis with the aim of identifying the molecular pathways associated with metabolic dysregulation during ASCI for the first time. We found a total of 51 pathways were enriched (Figure 5A), and further identified five pathways (purine metabolism, glycerophospholipid metabolism, nitrogen metabolism, glutathione metabolism, and ascorbate, and aldarate metabolism) with significant alteration based on DEGs number > 3, DEMs number > 3 and FDR < 0.01 (Table 6). Among these, purine metabolism is most significantly dysregulated at the gene and metabolite levels. A total of 48 DEGs and 16 DEMs were involved in the dysregulation of purine metabolism, of which 20 genes (Rrm2, Gucy2c, Nme4, Xdh, Nme6, Pnp, Ak2, Gda, Adcy4, Nt5c, Rrm1, Aprt, Entpd1, Nme2, Nt5e, Adss1, LOC314140, Impdh2, Adcy7, and Atic) and eight metabolites [xanthine, L-glutamine, deoxyinosine, xanthosine, inosine, ribose 1-phosphate, hypoxanthine, and 2’-deoxyinosine-5’-diphosphate (dIDP)] were significantly upregulated and 28 genes (Entpd4, Entpd5, Pde2a, Ampd2, Pde8b, Enpp4, Fhit, Adcy5, Ak4, Pde4c, Adcy9, Gucy1a1, Gucy2d, Entpd6, Pde4a, Adcy2, Adcy3, Ak7, Adcy1, Adcy8, Ak9, Gucy1b1, Pde1b, Gucy1a2, Ak5, Pde10a, Entpd3, and Pde11a) and eight [adenosine monophosphate (AMP), inosinic acid (IMP), adenosine, guanosine monophosphate (GMP), adenine, 5-aminoimidazole ribonucleotide (AIR), deoxyguanosine and 2’-deoxyguanosine-5’-diphosphate (dGDP)] metabolites were significantly downregulated (T). To visualize the expression levels and relationships of each DEG and DEM in purine metabolism, we have plotted them in the KEGG pathway (Figure 5B), and it is clear that these genes and metabolites play an important role in the dysregulation of purine metabolism during ASCI.

**TABLE 6:** Integrated pathway analysis of transcriptomic and metabolomic data.

Pathway name	*P*_adj_	FDR	Impact
Purine metabolism	1.33E−05	1.33E−05	1.503
Glycerophospholipid metabolism	2.42E−04	8.92E−05	0.941
Nitrogen metabolism	0.001	1.41E−04	0.778
Glutathione metabolism	0.001	3.03E−04	1.036
Phosphatidylinositol signaling system	0.021	0.004	0.781
Ascorbate and aldarate metabolism	0.061	0.009	1.125

## Discussion

**FIGURE 6:**
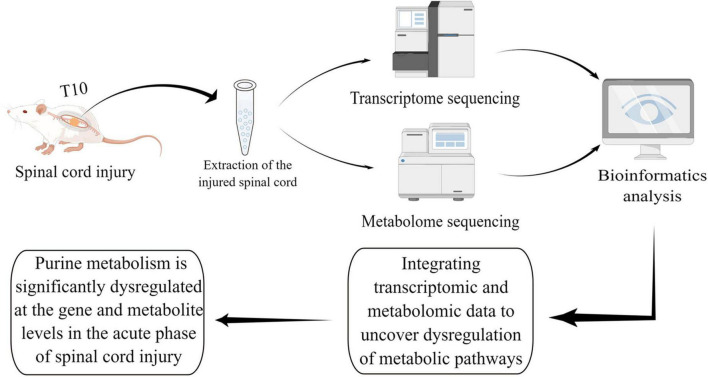
Schematic diagram of this study.

Spinal cord injury leads to substantial dysregulation of the metabolism in the injured microenvironment, which severely affects the recovery of neural tissue. Yet a systematic understanding of metabolic dysregulation in the SCI microenvironment is still lacking (Fan et al., 2022). In this study, we systematically explored the possible molecular mechanisms of metabolic dysregulation in the acute phase of SCI for the first time by integrating transcriptomic and metabolomic data. We found that several metabolic pathways were strikingly altered at both the gene and metabolite levels, with purine metabolism being the most striking (Figure 6, by Figdraw).

As one of the tissues with high metabolic demands, neural tissue requires a high metabolic rate to ensure biological processes such as axonal elongation, synaptic transmission, and action potential generation (Laughlin et al., 1998; Hallermann et al., 2012; Dolci et al., 2022). After SCI, the native metabolic processes are disrupted and the function of cells in the injured microenvironment is severely lost (LoPachin et al., 1999; Fujieda et al., 2012), resulting in a heavy impediment to the recovery of the spinal cord. We found that the tissue and cells of the injured microenvironment were markedly altered after SCI with increased necrotic tissue and decreased neurons, and subsequent transcriptomics and metabolomics analyses revealed that the injured microenvironment was also markedly dysregulated at the gene and metabolite levels.

Fujieda et al. (2012) found that more than 25% of metabolites in the injured microenvironment were significantly altered after SCI in rats using untargeted metabolomics analysis. In this study, we found that a total of 360 metabolites were altered in the injured microenvironment during the acute phase of rats with SCI, of which 310 were significantly upregulated, such as pantothenic acid, 2-ketobutyric acid, L-methionine, guanidoacetic acid, sphingosine, indoxyl sulfate, etc., and 50 were significantly downregulated, such as N6-acetyl-L-lysine, N-a-acetyl-L-arginine, oxoadipic acid, phosphocreatine, nicotinic acid mononucleotide, etc. Further analysis by KEGG showed that these DEMs are mainly involved in amino acid metabolism, neurotransmitter metabolism, nucleotide metabolism, and so on, which are necessary to maintain normal cellular functions. Previous studies have shown that the citric acid cycle could be dramatically impaired after SCI (Dolci et al., 2022). Coenzyme A, a key cofactor in the citric acid cycle, cannot be synthesized without pantothenic acid (Peterson et al., 2020). We found a significant increase in the expression of pantothenic acid after SCI, which may suggest that the synthesis of coenzyme A was suppressed and that the citric acid cycle of the injured microenvironment was affected. Methionine, an essential amino acid in mammals, is a metabolic precursor of 2-ketobutyric acid and cysteine (Martinez et al., 2017). Under normal conditions, 2-ketobutyric acid can be further converted to succinyl coenzyme A (Wang Y. et al., 2019), which can enter the citric acid cycle for energy supply; and cysteine is involved in the synthesis of glutathione thus exerting an antioxidant effect (Asantewaa and Harris, 2021). The elevation of 2-ketobutyric acid and L-methionine after SCI may suggest that the antioxidant system and energy supply system of the injured microenvironment are impaired and have toxic effects on neurons. Guanidoacetic acid, synthesized *de novo* from glycine and arginine, is the natural precursor of creatine in vertebrates (Ostojic et al., 2020). Normally, creatine can form phosphocreatine under the action of creatine kinase and then participate in the production of adenosine triphosphate (ATP) for energy supply (Kazak and Cohen, 2020). A recent metabolomic study of the injured spinal cord in rats showed increased levels of guanidoacetic acid in the acute phase of SCI (Yang et al., 2022). In the present study, we found that guanidoacetic acid expression was also elevated, and arginine and phosphocreatine expression were decreased in the acute phase of SCI, suggesting that creatine production from guanidoacetic acid was blocked and ATP production was reduced.

In addition to global metabolite alterations, our transcriptome profiling also identified significant changes at the gene level during the acute phase of SCI. In this study, we found that there were 5,963 DEGs in the ASCI group compared to the sham group, of which 2,848 genes were upregulated, and 3,115 genes were downregulated. Subsequent GO analysis revealed that these DEGs were mainly involved in biological processes such as responses to stimuli, immune system processes, and metabolic processes, which were similar findings to those of previous related studies (Shi et al., 2017; Gong et al., 2020). Particularly, we further analyzed the genes involved in metabolic processes and found that many of the upregulated genes were involved in nucleoside and nucleotide metabolic processes, ROS metabolic processes, and many of the downregulated genes were involved in nucleotide metabolic processes and neurotransmitter metabolic processes. As an important event in secondary SCI, the massive production of ROS not only exacerbates the damage of the local microenvironment but also causes neuronal damage in the upstream brain regions, which seriously hinders the recovery of neural tissue. Our previous study showed that SCI accelerates hippocampal oxidative stress, leading to neuronal mitochondrial ROS damage, resulting in reduced dendritic complexity, and synaptic communication, which may ultimately lead to cognitive dysfunction (Hu et al., 2022). A recent study has shown that as a member of the hexokinases (Hks) family, Hk3 deficiency increases intracellular ROS, leading to increased damage to cellular DNA and impaired DNA repair (Seiler et al., 2022). Ncf2 is a core subunit of the nicotinamide adenine dinucleotide phosphate oxidase complex that produces superoxide to exacerbate oxidative stress (Mo et al., 2019). We found a significant increase in the expression of Hk3 and Ncf2 after SCI, which may suggest that the balance of the endogenous oxidative-antioxidant system was disrupted, and their values in SCI warrant further investigation in the future. Neuronal calcium sensor (Ncs) 1, a member of the intracellular Ncs family, encodes a high-affinity intracellular calcium-binding protein that is expressed in most neural tissues. Under normal conditions, Ncs1 can release calcium-controlled neurotransmitters into neurons through the regulation of voltage-gated calcium channel and can also act as a regulator promoting neurite outgrowth (Bandura and Feng, 2019). In the present study, the expression of Ncs1 was significantly downregulated, which may suggest that the release of calcium-controlled neurotransmitters and neurite outgrowth were blocked.

By using single transcriptomics and metabolomics analysis, we found that the transcriptome and metabolome profiles were highly changed in ASCI. However, due to the complexity of the biological system and the limitation of each omics platform, a single omics is inadequate to explain the pathological mechanism of the disease, and therefore there is a need to integrate transcriptomic and metabolomic data to reveal more biological insights into the disease. Currently, there is no study on integrating transcriptomics and metabolomics analysis to investigate ASCI, thus we further performed an integration analysis based on pathway to deeply reveal the underlying mechanisms of ASCI metabolic disturbances. Surprisingly, we found five pathways significantly altered based on DEGs number > 3, DEMs number > 3 and FDR < 0.01, with the purine metabolism pathway being the most significantly dysregulated at the gene and metabolite levels, involving a total of 16 DEMs and 48 DEGs.

As the most abundant metabolites, purines include adenine nucleotides (ATP, ADP, AMP) and related molecules such as cAMP, NAD, adenosine, hypoxanthine and adenine, and also guanine nucleotides (GTP, GDP, GMP) as well as cGMP, guanosine, guanine and xanthine (Gottle et al., 2013). They are not only essential for nucleotide synthesis, but also for cell survival and proliferation, serving as indispensable small organic molecules for cells. Purine metabolism maintains the cellular pool of adenosine and guanosine acids through the synthesis and degradation of purine nucleotides. There are two different pathways for purine nucleotide synthesis in mammalian cells: the salvage pathway and the *de novo* biosynthetic pathway (Yin et al., 2018). The synthesis of purines in spinal cord tissues relies mainly on the salvage pathway due to the lack of relevant enzymatic reactions for the *de novo* biosynthetic pathway in neural tissues (Ipata, 2011). The salvage pathway of purines provides purine nucleotide source by recycling degraded bases, mainly including AMP, IMP, GMP, adenine and adenosine (Huang et al., 2021). The degradation pathway of purines mainly involves inosine, hypoxanthine, xanthine, and xanthosine, where hypoxanthine can be converted to IMP by enzymatic reactions (Yin et al., 2018). In the present study, we found that the levels of AMP, IMP, GMP, adenine and adenosine were significantly decreased, while the levels of inosine, hypoxanthine, xanthine, and xanthosine were significantly increased, which may suggest dysregulation of purine synthesis in the injured spinal cord microenvironment. A recent study found that purine depletion stimulated cell migration despite effectively reducing cell proliferation (Soflaee et al., 2022). In central nervous system injury, purine release contributes to triggering astrogliosis (Illes et al., 2016), and the reduction of purines in the acute phase of SCI may adversely affect astrocyte proliferation, which requires further study in the future.

**FIGURE 7:**
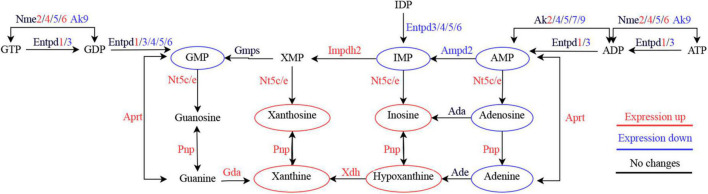
Simplified diagram of purine metabolism dysregulation in the acute phase of spinal cord injury.

Meanwhile, at the gene level, we found significant upregulation of genes nucleoside diphosphate kinase (Nme) 2, Nme4, Nme6, purine nucleoside phosphorylase (Pnp), adenine phosphoribosyl transferase (Aprt), xanthine dehydrogenase (Xdh), guanine deaminase (Gda) and adenylate kinase (Ak) 2, and significant downregulation of genes Ak4, Ak5, Ak7, Ak9, ectonucleoside triphosphate diphosphohydrolase (Entpd) 3, Entpd4, Entpd5, Entpd6, and adenosine monophosphate deaminase (Ampd) 2. The proteins encoded by these genes play an important sway in the salvage biosynthetic pathway and degradation pathway of purines. The Nme family is associated with the phosphorylation of nucleotide diphosphates to form nucleotide triphosphates (Garcia-Esparcia et al., 2015). Pnp can reversibly catalyze the phosphorylated hydrolysis of purine nucleosides. Aprt is related to adenine metabolism and catalyzes phosphorylation reactions. Xdh is involved in the degradation of purines and acts as one of the main sources of ROS in the tissue oxidative stress response (Kushiyama et al., 2014). Gda catalyzes the deamination of guanine to xanthine, an important component of the guanine degradation pathway (Wang J. et al., 2019). The elevated expression levels of both Xdh and Gda in the injured spinal cord microenvironment may suggest an exacerbation of oxidative stress. The Ak family is involved in AMP phosphorylation to ADP and dAMP phosphorylation to dATP, which play a variety of intra- and extracellular functions such as regulation of nuclear transport, DNA synthesis and repair, and energy metabolism (Dzeja and Terzic, 2009). The Entpd family is known to hydrolyze ATP or ADP to AMP with different substrate preferences (Junger, 2011), and Ampd2 is the hepatic isoenzyme of three known AMP deaminases that convert AMP to IMP and is essential for purine metabolism (Van den Bergh and Sabina, 1995). The reduced expression levels of Aks, Entpds and Ampd2 may help to explain the reduction of AMP and IMP in purine metabolism in the injured microenvironment. Overall, integrative biology showed marked deviations in purine metabolism at the gene and metabolite levels (Figure 7).

In line with other single omics, our transcriptomics and metabolomics results showed significant changes in genes and metabolites during ASCI. Although we have identified key hub genes and metabolites in the dysregulation of purine metabolism during ASCI by combining the results of transcriptomics and metabolomics, and the role of these genes and metabolites in the pathophysiology of SCI requires further investigation due to the limitations of this study, such as the small sample size and the difference of each single omics platform.

In summary, for the first time, we revealed remarkable dysregulation of purine metabolism at both transcriptome and metabolome levels in the acute phase of SCI in rats by integrating metabolomic and transcriptomic profiles of the injured spinal cord, which could seriously affect the energy metabolism of the local microenvironment and increase oxidative stress as well as other responses detrimental to nerve repair and regeneration. These findings may contribute to a better understanding of the pathological mechanisms in the acute phase of spinal cord injury and also help to shift the treatment of spinal cord injury from a single-element to a multi-element, complex networked repair approach.

## Data availability statement

The datasets presented in this study can be found in online repositories. The names of the repository/repositories and accession number(s) can be found below: https://www.ncbi.nlm.nih.gov/, PRJNA895603; https://www.ebi.ac.uk/metabolights/, MTBLS6350.

## Ethics statement

The animal study was reviewed and approved by Animal Research Ethics Committee of Ningxia Medical University.

## Author contributions

ZZ and HX: conceived and designed the study. ZZ, ML, ZJ, and YL: conducted the experiments. LC and YC: analyzed the data. ZZ and HL: prepared the figures. HX and XH: supervised the study. ZZ, JH, and LZ: wrote the manuscript. All authors contributed to the article and approved the submitted version.

## Footnotes

1
https://www.metaboanalyst.ca/

## References

[B1] Ahuja C. S. Fehlings M. (2016). Concise review: Bridging the gap: Novel neuroregenerative and neuroprotective strategies in spinal cord injury. *Stem. Cells Transl. Med.* 5 914–924. 10.5966/sctm.2015-0381 27130222 PMC4922857

[B2] Ahuja C. S. Wilson J. R. Nori S. Kotter M. R. N. Druschel C. Curt A. (2017). Traumatic spinal cord injury. *Nat. Rev. Dis. Primers* 3:17018. 10.1038/nrdp.2017.18 28447605

[B3] Alizadeh A. Dyck S. M. Karimi-Abdolrezaee S. (2019). Traumatic spinal cord injury: An overview of pathophysiology, models and acute injury mechanisms. *Front. Neurol.* 10:282. 10.3389/fneur.2019.00282 30967837 PMC6439316

[B4] Anjum A. Yazid M. D. Fauzi Daud M. Idris J. Ng A. M. H. Selvi Naicker A. (2020). Spinal cord injury: Pathophysiology, multimolecular interactions, and underlying recovery mechanisms. *Int. J. Mol. Sci.* 21:7533. 10.3390/ijms21207533 33066029 PMC7589539

[B5] Asantewaa G. Harris I. S. (2021). Glutathione and its precursors in cancer. *Curr. Opin. Biotechnol.* 68 292–299. 10.1016/j.copbio.2021.03.001 33819793

[B6] Bandura J. Feng Z. P. (2019). Current understanding of the role of neuronal calcium sensor 1 in neurological disorders. *Mol. Neurobiol.* 56 6080–6094. 10.1007/s12035-019-1497-2 30719643

[B7] Bansal N. Kumar M. Sankhwar S. N. Gupta A. (2022). Relevance of emerging metabolomics-based biomarkers of prostate cancer: A systematic review. *Expert Rev. Mol. Med.* 24:e25. 10.1017/erm.2022.20 35730322

[B8] Baroncini A. Maffulli N. Eschweiler J. Tingart M. Migliorini F. (2021). Pharmacological management of secondary spinal cord injury. *Expert Opin. Pharmacother.* 22 1793–1800. 10.1080/14656566.2021.1918674 33899630

[B9] Basso D. M. Beattie M. S. Bresnahan J. C. (1995). A sensitive and reliable locomotor rating scale for open field testing in rats. *J Neurotrauma* 12 1–21. 10.1089/neu.1995.12.1 7783230

[B10] Bradbury E. J. Burnside E. R. (2019). Moving beyond the glial scar for spinal cord repair. *Nat. Commun.* 10:3879. 10.1038/s41467-019-11707-7 31462640 PMC6713740

[B11] Chu X. Zhang B. Koeken V. Gupta M. K. Li Y. (2021). Multi-omics approaches in immunological research. *Front. Immunol.* 12:668045. 10.3389/fimmu.2021.668045 34177908 PMC8226116

[B12] di Meo N. A. Loizzo D. Pandolfo S. D. Autorino R. Ferro M. Porta C. (2022). Metabolomic approaches for detection and identification of biomarkers and altered pathways in bladder cancer. *Int. J. Mol. Sci.* 23:4173. 10.3390/ijms23084173 35456991 PMC9030452

[B13] Dolci S. Mannino L. Bottani E. Campanelli A. Di Chio M. Zorzin S. (2022). Therapeutic induction of energy metabolism reduces neural tissue damage and increases microglia activation in severe spinal cord injury. *Pharmacol. Res.* 178:106149. 10.1016/j.phrs.2022.106149 35240272

[B14] Dzeja P. Terzic A. (2009). Adenylate kinase and AMP signaling networks: Metabolic monitoring, signal communication and body energy sensing. *Int. J. Mol. Sci.* 10 1729–1772. 10.3390/ijms10041729 19468337 PMC2680645

[B15] Fan B. Wei Z. Feng S. (2022). Progression in translational research on spinal cord injury based on microenvironment imbalance. *Bone Res.* 10:35. 10.1038/s41413-022-00199-9 35396505 PMC8993811

[B16] Fan B. Wei Z. Yao X. Shi G. Cheng X. Zhou X. (2018). Microenvironment imbalance of spinal cord injury. *Cell Transplant.* 27 853–866. 10.1177/0963689718755778 29871522 PMC6050904

[B17] Figueroa J. D. De Leon M. (2014). Neurorestorative targets of dietary long-chain omega-3 fatty acids in neurological injury. *Mol. Neurobiol.* 50 197–213. 10.1007/s12035-014-8701-1 24740740 PMC4183712

[B18] Fujieda Y. Ueno S. Ogino R. Kuroda M. Jonsson T. J. Guo L. (2012). Metabolite profiles correlate closely with neurobehavioral function in experimental spinal cord injury in rats. *PLoS One* 7:e43152. 10.1371/journal.pone.0043152 22912814 PMC3418274

[B19] Gao C. Shen X. Tan Y. Chen S. (2022). Pathogenesis, therapeutic strategies and biomarker development based on “omics” analysis related to microglia in Alzheimer’s disease. *J. Neuroinflammation* 19:215. 10.1186/s12974-022-02580-1 36058959 PMC9441025

[B20] Garcia-Esparcia P. Hernandez-Ortega K. Ansoleaga B. Carmona M. Ferrer I. (2015). Purine metabolism gene deregulation in Parkinson’s disease. *Neuropathol. Appl. Neurobiol.* 41 926–940. 10.1111/nan.12221 25597950

[B21] Gong L. Lv Y. Li S. Feng T. Zhou Y. Sun Y. (2020). Changes in transcriptome profiling during the acute/subacute phases of contusional spinal cord injury in rats. *Ann. Transl. Med.* 8:1682. 10.21037/atm-20-6519 33490194 PMC7812200

[B22] Gottle M. Burhenne H. Sutcliffe D. Jinnah H. A. (2013). Purine metabolism during neuronal differentiation: The relevance of purine synthesis and recycling. *J. Neurochem.* 127 805–818. 10.1111/jnc.12366 23859490 PMC3859826

[B23] Hachem L. D. Ahuja C. S. Fehlings M. G. (2017). Assessment and management of acute spinal cord injury: From point of injury to rehabilitation. *J. Spinal Cord Med.* 40 665–675. 10.1080/10790268.2017.1329076 28571527 PMC5778930

[B24] Hallermann S. de Kock C. P. Stuart G. J. Kole M. H. (2012). State and location dependence of action potential metabolic cost in cortical pyramidal neurons. *Nat. Neurosci.* 15 1007–1014. 10.1038/nn.3132 22660478

[B25] Hu X. Wu L. Wen Y. Liu J. Li H. Zhang Y. (2022). Hippocampal mitochondrial abnormalities induced the dendritic complexity reduction and cognitive decline in a rat model of spinal cord injury. *Oxid. Med. Cell. Longev.* 2022:9253916. 10.1155/2022/9253916 35571236 PMC9095360

[B26] Huang Z. Xie N. Illes P. Di Virgilio F. Ulrich H. Semyanov A. (2021). From purines to purinergic signalling: Molecular functions and human diseases. *Signal Transduct. Target. Ther.* 6:162. 10.1038/s41392-021-00553-z 33907179 PMC8079716

[B27] Illes P. Verkhratsky A. Burnstock G. Sperlagh B. (2016). Purines in neurodegeneration and neuroregeneration. *Neuropharmacology* 104 1–3. 10.1016/j.neuropharm.2016.01.020 26775822

[B28] Ipata P. L. (2011). Origin, utilization, and recycling of nucleosides in the central nervous system. *Adv. Physiol. Educ.* 35 342–346. 10.1152/advan.00068.2011 22139768

[B29] Jiang H. Peng J. Zhou Z. Y. Duan Y. Chen W. Cai B. (2010). Establishing (1)H nuclear magnetic resonance based metabonomics fingerprinting profile for spinal cord injury: A pilot study. *Chin. Med. J. (Engl).* 123 2315–2319. 21034541

[B30] Junger W. G. (2011). Immune cell regulation by autocrine purinergic signalling. *Nat. Rev. Immunol.* 11 201–212. 10.1038/nri2938 21331080 PMC4209705

[B31] Karahalil B. (2016). Overview of systems biology and omics technologies. *Curr. Med. Chem.* 23 4221–4230. 10.2174/0929867323666160926150617 27686657

[B32] Kazak L. Cohen P. (2020). Creatine metabolism: Energy homeostasis, immunity and cancer biology. *Nat. Rev. Endocrinol.* 16 421–436. 10.1038/s41574-020-0365-5 32493980

[B33] Kushiyama A. Tanaka K. Hara S. Kawazu S. (2014). Linking uric acid metabolism to diabetic complications. *World J. Diabetes* 5 787–795. 10.4239/wjd.v5.i6.787 25512781 PMC4265865

[B34] Laughlin S. B. de Ruyter van Steveninck R. R. Anderson J. C. (1998). The metabolic cost of neural information. *Nat. Neurosci.* 1 36–41. 10.1038/236 10195106

[B35] Lindsay S. L. McCanney G. A. Willison A. G. Barnett S. C. (2020). Multi-target approaches to CNS repair: Olfactory mucosa-derived cells and heparan sulfates. *Nat. Rev. Neurol.* 16 229–240. 10.1038/s41582-020-0311-0 32099190

[B36] LoPachin R. M. Gaughan C. L. Lehning E. J. Kaneko Y. Kelly T. M. Blight A. (1999). Experimental spinal cord injury: Spatiotemporal characterization of elemental concentrations and water contents in axons and neuroglia. *J. Neurophysiol.* 82 2143–2153. 10.1152/jn.1999.82.5.2143 10561394

[B37] Martinez Y. Li X. Liu G. Bin P. Yan W. Mas D. (2017). The role of methionine on metabolism, oxidative stress, and diseases. *Amino Acids* 49 2091–2098. 10.1007/s00726-017-2494-2 28929442

[B38] Mathew A. V. Jaiswal M. Ang L. Michailidis G. Pennathur S. Pop-Busui R. (2019). Impaired amino acid and TCA metabolism and cardiovascular autonomic neuropathy progression in type 1 diabetes. *Diabetes* 68 2035–2044. 10.2337/db19-0145 31337616 PMC6754246

[B39] Mo X. G. Liu W. Yang Y. Imani S. Lu S. Dan G. (2019). NCF2, MYO1F, S1PR4, and FCN1 as potential noninvasive diagnostic biomarkers in patients with obstructive coronary artery: A weighted gene co-expression network analysis. *J. Cell Biochem.* 120 18219–18235. 10.1002/jcb.29128 31245869 PMC6771964

[B40] Olivier M. Asmis R. Hawkins G. A. Howard T. D. Cox L. A. (2019). The need for multi-omics biomarker signatures in precision medicine. *Int. J. Mol. Sci.* 20:4781. 10.3390/ijms20194781 31561483 PMC6801754

[B41] O’Shea T. M. Burda J. E. Sofroniew M. V. (2017). Cell biology of spinal cord injury and repair. *J. Clin. Invest.* 127 3259–3270. 10.1172/JCI90608 28737515 PMC5669582

[B42] Ostojic S. M. Ratgeber L. Olah A. Betlehem J. Acs P. (2020). Guanidinoacetic acid deficiency: A new entity in clinical medicine? *Int. J. Med. Sci.* 17 2544–2550. 10.7150/ijms.47757 33029096 PMC7532483

[B43] Peng J. Zeng J. Cai B. Yang H. Cohen M. J. Chen W. (2014). Establishment of quantitative severity evaluation model for spinal cord injury by metabolomic fingerprinting. *PLoS One* 9:e93736. 10.1371/journal.pone.0093736 24727691 PMC3984092

[B44] Peterson C. T. Rodionov D. A. Osterman A. L. Peterson S. N. (2020). B vitamins and their role in immune regulation and cancer. *Nutrients* 12:3380. 10.3390/nu12113380 33158037 PMC7693142

[B45] Rodgers H. M. Patton R. Yow J. Zeczycki T. N. Kew K. Clemens S. (2022). Morphine resistance in spinal cord injury-related neuropathic pain in rats is associated with alterations in dopamine and dopamine-related metabolomics. *J. Pain* 23 772–783. 10.1016/j.jpain.2021.11.009 34856409

[B46] Seiler K. Humbert M. Minder P. Mashimo I. Schlafli A. M. Krauer D. (2022). Hexokinase 3 enhances myeloid cell survival via non-glycolytic functions. *Cell Death Dis.* 13:448. 10.1038/s41419-022-04891-w 35538058 PMC9091226

[B47] Shi L. L. Zhang N. Xie X. M. Chen Y. J. Wang R. Shen L. (2017). Transcriptome profile of rat genes in injured spinal cord at different stages by RNA-sequencing. *BMC Genomics* 18:173. 10.1186/s12864-017-3532-x 28201982 PMC5312572

[B48] Soflaee M. H. Kesavan R. Sahu U. Tasdogan A. Villa E. Djabari Z. (2022). Purine nucleotide depletion prompts cell migration by stimulating the serine synthesis pathway. *Nat. Commun.* 13:2698. 10.1038/s41467-022-30362-z 35577785 PMC9110385

[B49] Sullivan P. G. Krishnamurthy S. Patel S. P. Pandya J. D. Rabchevsky A. G. (2007). Temporal characterization of mitochondrial bioenergetics after spinal cord injury. *J. Neurotrauma* 24 991–999. 10.1089/neu.2006.0242 17600515

[B50] Van den Bergh F. Sabina R. L. (1995). Characterization of human AMP deaminase 2 (AMPD2) gene expression reveals alternative transcripts encoding variable N-terminal extensions of isoform L. *Biochem. J.* 312(Pt 2) 401–410. 10.1042/bj3120401 8526848 PMC1136276

[B51] Wang Y. Bi C. Pang W. Liu Y. Yuan Y. Zhao H. (2019). Plasma metabolic profiling analysis of gout party on acute gout arthritis rats based on UHPLC-Q-TOF/MS combined with multivariate statistical analysis. *Int. J. Mol. Sci.* 20:5753. 10.3390/ijms20225753 31731809 PMC6888674

[B52] Wang J. Bing T. Zhang N. Shen L. He J. Liu X. (2019). The mechanism of the selective antiproliferation effect of guanine-based biomolecules and its compensation. *ACS Chem. Biol.* 14 1164–1173. 10.1021/acschembio.9b00062 31083967

[B53] Wu Y. Streijger F. Wang Y. Lin G. Christie S. Mac-Thiong J. M. (2016). Parallel metabolomic profiling of cerebrospinal fluid and serum for identifying biomarkers of injury severity after acute human spinal cord injury. *Sci. Rep.* 6:38718. 10.1038/srep38718 27966539 PMC5155264

[B54] Yang H. Zhang P. Xie M. Luo J. Zhang J. Zhang G. (2022). Parallel metabolomic profiling of cerebrospinal fluid, plasma, and spinal cord to identify biomarkers for spinal cord injury. *J. Mol. Neurosci.* 72 126–135. 10.1007/s12031-021-01903-w 34498202 PMC8755701

[B55] Yang L. Y. Tsai M. Y. Juan S. H. Chang S. F. Yu C. R. Lin J. C. (2021). Exerting the appropriate application of methylprednisolone in acute spinal cord injury based on time course transcriptomics analysis. *Int. J. Mol. Sci.* 22:13024. 10.3390/ijms222313024 34884829 PMC8657964

[B56] Yao X. Q. Liu Z. Y. Chen J. Y. Huang Z. C. Liu J. H. Sun B. H. (2021). Proteomics and bioinformatics reveal insights into neuroinflammation in the acute to subacute phases in rat models of spinal cord contusion injury. *FASEB J.* 35:e21735. 10.1096/fj.202100081RR 34143440

[B57] Yin J. Ren W. Huang X. Deng J. Li T. Yin Y. (2018). Potential mechanisms connecting purine metabolism and cancer therapy. *Front. Immunol.* 9:1697. 10.3389/fimmu.2018.01697 30105018 PMC6077182

